# Pilot Study of Real-World Monitoring of the Heart Rate Variability in Amyotrophic Lateral Sclerosis

**DOI:** 10.3389/frai.2022.910049

**Published:** 2022-07-07

**Authors:** Alexander A. Brown, Bradley J. Ferguson, Vovanti Jones, Bruce E. Green, Justin D. Pearre, Ifeoma A. Anunoby, David Q. Beversdorf, Richard J. Barohn, Carmen M. Cirstea

**Affiliations:** ^1^Department of Psychological Sciences, College of Arts and Science, University of Missouri, Columbia, MO, United States; ^2^Department of Health Psychology, School of Health Professions, University of Missouri, Columbia, MO, United States; ^3^Department of Radiology, School of Medicine, University of Missouri, Columbia, MO, United States; ^4^Department of Physical Medicine and Rehabilitation, School of Medicine, University of Missouri, Columbia, MO, United States; ^5^School of Medicine, University of Missouri, Columbia, MO, United States; ^6^College of Arts and Science, University of Missouri, Columbia, MO, United States; ^7^Department of Neurology, School of Medicine, University of Missouri, Columbia, MO, United States

**Keywords:** amyotrophic lateral sclerosis, heart rate variability, autonomic nervous system, Vista solution, magnetic resonance imaging, insula

## Abstract

**Aims:**

Cardiovascular dysautonomia may impact the quality of life and survival in amyotrophic lateral sclerosis (ALS). Such dysfunction is not systematically assessed in these patients. Wearable devices could help. The feasibility of a wearable biosensor to detect heart rate variability (HRV), a physiological marker of sympathovagal balance, was studied for the first time in real-world settings in ALS.

**Methods:**

Five ALS patients (two early/three late; one bulbar-onset; mildly-to-moderately disabled) and five age/sex/BMI/comorbidities-matched controls underwent assessment of 3-day HRV *via* VitalConnect biosensor (worn on the left thorax). De-identified data captured by the biosensor were transferred to a secure cloud server *via* a relay Bluetooth device. Baseline ALS severity/anxiety and physical activity during testing were documented/quantified. Time-domain HRV measures (i.e., pNN50) were analyzed.

**Results:**

An overall 3-day abnormal HRV (pNN50 < 3%), was found in three out of five patients (mean ± SD for the group, 2.49 ± 1.51). Similar changes were reported in controls (12.32 ± 21.14%). There were no statistically significant relationships between pNN50 values and baseline anxiety or physical activity during the tested days (*p* > 0.05 for both groups). A negative correlation was found between pNN50 values and age in patients (*p* = 0.01) and controls (*p* = 0.09), which is similar with what is found in the general population. In line with prior studies, pNN50 values were independent of disease stage (*p* = 0.6) and disability (*p* = 0.4).

**Conclusions:**

These preliminary results suggest that remote HRV measures using the VitalConnect is feasible and may constitute an improved strategy to provide insights into sympathovagal balance in ALS. Further work with larger sample sizes is warranted.

## Introduction

Precision medicine, which emphasizes leveraging all available data about an individual to improve health outcomes, is in its nascence in amyotrophic lateral sclerosis (ALS). Despite dozens of clinical trials, no new advances in treatment have been brought to this vulnerable population. One reason behind this lack of progress is an incomplete understanding of the basic elements of ALS neurophysiopathology. For example, in addition to classical motor impairments, dysfunction of the autonomic nervous system (ANS), also known as dysautonomia, may occur during ALS regardless of the disease stage (Oey et al., 2002; Shindo et al., 2004; Baltadzhieva et al., 2005; Pavlovic et al., 2010; Merico and Cavinato, 2011; Pinto et al., 2012; Pimentel et al., 2019). Converging evidence indicates that such dysfunction may worsen the quality of life and influence survival, particularly in the advanced stages of disease (Baltadzhieva et al., 2005; Asai et al., 2007; Merico and Cavinato, 2011; Pinto et al., 2012; Pimentel et al., 2019). Symptoms of dysautonomia are variable and include cardiovascular symptoms, gastrointestinal, urogenital, sudomotor, and thermoregulatory dysfunction, pupillary abnormalities, and sleep and respiratory disorders. In early ALS, these symptoms are subclinical in most patients. Likely, current clinical testing doesn't sufficiently capture the individual symptom constellation and pathogenesis. Thus, critical information is lost, impeding precision medicine in this disease. State-of-the-art devices could help, and based on our extensive experience in examining autonomic functioning in clinical populations (Ferguson et al., 2016, 2017; Zamzow et al., 2016), the main goal of the current study was to use a new device approved by the US Food and Drug Administration for wireless remote monitoring of vital signs (Vital Connect, Vista solution) to quantify continuous and unobstructed cardiovagal function in real-world settings in patients with ALS. Heart rate variability (HRV) is a widely-used physiological marker of sympathovagal balance (Katona et al., 1970; Camm Aj et al., 1996) that can be reliably measured in real-world settings. Considering the progressive and debilitating nature of this disease restricting patients' travel for testing, we used for the first time the Vista solution to collect HRV for 3 consecutive days in ALS patients in their natural environment.

Prior evidence shows more predominant cardiovagal dysfunction in bulbar-onset patients than in those with spinal onset (Oey et al., 2002; Shindo et al., 2004; Merico and Cavinato, 2011). Specifically, higher resting heart rate and blood pressure and lower heart rate variability were found in these patients compared to healthy controls (Linden et al., 1998; Oey et al., 2002). Critically, these dysfunctions were not related to the clinical features or disease duration (Linden et al., 1998; Pavlovic et al., 2010; Merico and Cavinato, 2011), suggesting that dysautonomia in early ALS could be a primary phenomenon, paralleling the degeneration of motor neurons. Plasma norepinephrine studies provide further support for this theory; levels of norepinephrine were found elevated and not correlated with the extent of motor disability (Ziegler et al., 1980; Yamashita et al., 1997; Ohno et al., 2001). Despite this, there is a lack of neurobiologically driven research on dysautonomia in early ALS, leading to a lack of identified biomarkers and specific therapeutic targets. We, therefore, included bulbar- and spinal-onset patients in the early and late phases of the disease in the present study.

When recognized, dysautonomia can be treated, sometimes successfully, improving the quality of life in ALS patients. Patients in the most advanced stage of ALS frequently show severe autonomic symptoms, so-called autonomic storm (Shimizu et al., 1994, 1996). A limbic origin was suggested for such symptoms (Shimizu et al., 1994), although the precise pathophysiology remains to be elucidated. The insula is a crucial central autonomic area controlling both sympathetically and parasympathetically-mediated cardiovascular function (Colivicchi et al., 2005; Nagai et al., 2010; Oppenheimer and Cechetto, 2016; Sposato et al., 2016), and of direct relevance to this study, is vulnerable to ALS-related neurodegeneration (Wicks et al., 2008; Consonni et al., 2018). There are a few reports that showed notable relationships between insula neurodegeneration and low survival (Consonni et al., 2018) and cognitive, especially language, impairments (Wicks et al., 2008) in this population. There have been no previous studies investigating whether insular alterations provide additional information on autonomic dysfunction in any stage of the disease. Therefore, a secondary analysis was performed on these patients on the relationship between insular cortex structure (high-resolution structural MRI) and HRV metrics.

## Methods

The study was performed with the approval of the University of Missouri (MU) Human Subjects Review Board. All participants gave written informed consent before participation.

### Participants

Patients were recruited by several coauthors (VJ, JP, BG, IA) from the Muscular Dystrophy Association and Amyotrophic Lateral Sclerosis Clinics at MU. Patients were recruited if they received a diagnosis of sporadic or familial (genetically confirmed) ALS, ALS as probable or definitive (World Federation of Neurology revised El Escorial criteria), with bulbar or spinal-onset. Patients did not have any serious uncontrolled problems or were on confounding medications (opioids, anticholinergics). Five ALS patients have been recruited so far: two newly diagnosed patients (mean ± SD, 3.3 ± 0.2 mo after diagnosis) and three in late ALS (63.1 ± 5.3 mo); for all patients: age, 55.4 ± 15.0 years; two males; body mass index (BMI, 28.8 ± 2.9); two on riluzole, one on radicava; one bulbar-onset. Patient functional disability was assessed by the revised ALS Functional Rating Scale (ALSFRS-R) (Cedarbaum et al., 1999). At the time of the study, the patients had a total ALSFRS-R score from 25 to 44 (37.2 ± 7.3). Patients who could stand, walk without support, breathe spontaneously, and take meals regularly were included in the study. Those with a history of prolonged bed rest and/or immobility or diabetes mellitus were excluded. None of the participants had a history of respiratory disease. Since anxiety disorders are associated with impaired vagal function (reduced HRV) (Chalmers et al., 2014), anxiety was also measured (Beck Anxiety Inventory). At the time of the study, the patients had an anxiety score from 1 to 39 (14.0 ± 14.8). Five age/sex/BMI/comorbidities (hypertension/arthritis/baseline anxiety)-matched controls were recruited (*p* > 0.3 for all). All participants were free of alcohol or illicit drug use or MRI contraindications.

Patients and controls underwent HRV/clinical testing and MRI. This study is still ongoing; preliminary HRV data from 5 patients and 4 controls and MRI data from all participants were presented and discussed here.

### HRV Testing

**Figure 1:**
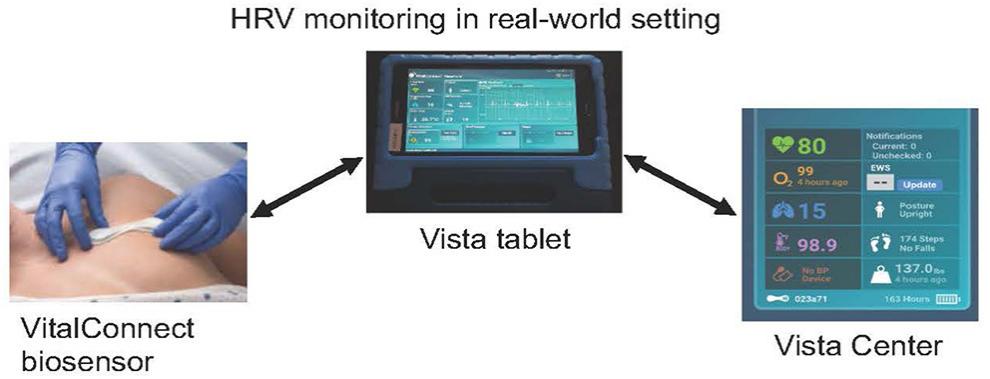
Vista solution platform comprised of (i) a VitalConnect biosensor (single-use, waterproof wireless medical device containing ECG electrodes, applied on the left thorax) that collects unobtrusively, objectively, and continuously 8 vital sign data, including heart rate, (ii) a tablet (Vista tablet, Bluetooth connected), a relay device for bi-directional communications with the biosensor, and (iii) a secured cloud server (Vista center), allowing robust data capture, transfer, and secure storage of encrypted, de-identified patient data.

The Vital Connect consists of a small, lightweight, waterproof, wireless, and disposable adhesive patch containing a biosensor (Figure 1). This biosensor unobtrusively, objectively, and continuously collects several metrics of vital sign data: HR, heart rate variability (HRV), RR, breathing rate, skin temperature, and the number of steps. The patch was attached to the patient's left thorax, from where the data are transmitted to a tablet (*via* Bluetooth, Vista tablet). The Vista tablet is a relay device for bi-directional communications with the biosensor and the Vista center (secured cloud server), allowing robust data capture, transfer, and secure storage of encrypted, de-identified patient data. The patch is powered by a coin-cell battery that lasts 7 days. This unique testing was used for the first time in patients with ALS.

This system was easily deployed in home settings. For continued communication between the biosensor and the tablet, the participant was instructed to keep the Vista tablet near their person. During the 3 days, a daily log, including the time and duration of each activity performed, was completed by the participant. For some patients residing outside of Columbia, Missouri, where the study took place, the tablet was return by mail with a prepaid envelop provided to the patient by the study team.

**Figure 2:**
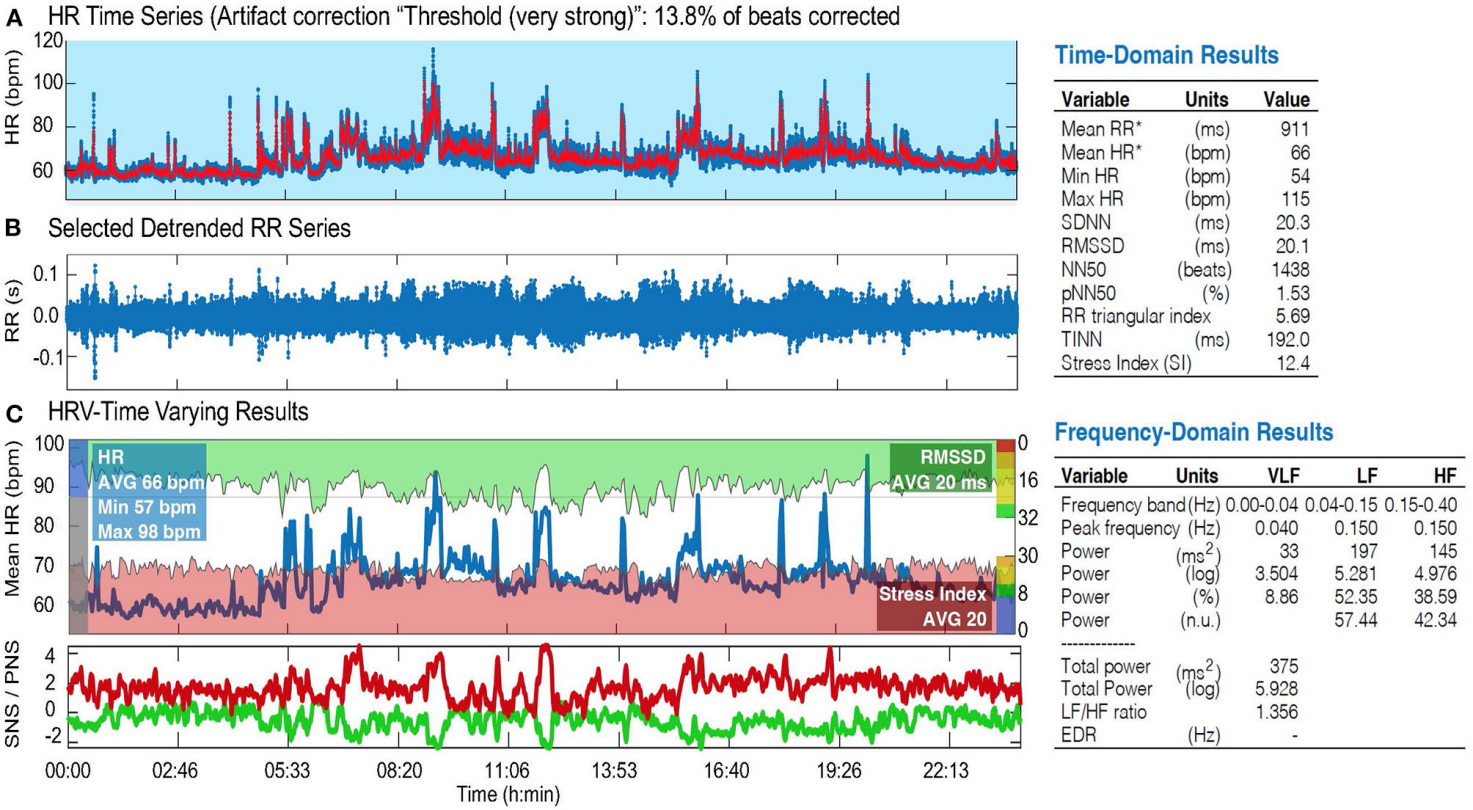
HRV analysis (Kubios) in one ALS patient showing altered sympathovagal imbalance: HRV time series **(A)**, selected detrended RR series **(B)**, and HRV-time varying results **(C)**, including time and frequency-domain results. HR, heart rate; SNS/PNS, sympathetic/parasympathetic nervous system; bpm, beats per minute; RMSSD, root mean square of the successive differences; SDNN, standard deviation of normal to normal R-R intervals; NN50, number of pairs of successive NN (R-R) intervals that differ by more than 50 ms; pNN50, proportion of NN50 divided by the total number of NN (R-R) intervals; TINN, triangular interpolation of NN interval histogram; VLF, very low frequency; LF, low frequency; HF, high frequency band of HRV.

HRV, step count, sleep hours, and breathing rate data were collected. Since this study is still an ongoing process, only HRV and steps count data were presented and discussed here. Of direct relevance to this study, the robustness of the ECG measurements with VitalConnect biosensor was recently demonstrated in hospitalized patients (Weenk et al., 2017, 2020; Stehlik et al., 2020). We analyzed ECG data using Kubios HRV Premium, Version 3.1.0.1 (Tarvainen et al., 2014). This analysis was detailed previously (Ferguson et al., 2017). Briefly, the RR interval data were visually inspected for artifacts, and those records with >20% of the beats corrected for artifacts were not analyzed. Critically, no records were discarded as the data from the VitalPatch did not reach the 20% threshold. Artifacts were corrected using the included artifact detection algorithm provided by Kubios HRV Premium (Lipponen and Tarvainen, 2019). For the purposes of this pilot study, only time-domain HRV measures were analyzed and discussed (Figure 2; Table 1). The main outcome was the percentage of pairs of consecutive RR intervals that differed by more than 50 ms or more, widely known as pNN50 (Bigger et al., 1988). A pNN50 <3% is considered abnormal and is reflective of sympathovagal imbalance. Our team has also explored other parameters from the time-domain (e.g., RR interval and square root of the mean squared differences of successive normal-to-normal RR intervals) (Figure 2) as they provide a wealth of information regarding sympathetic and parasympathetic changes. Each recording lasted 3 consecutive days and the averaged pNN50 for the entire recording period was presented and discussed here We also conducted a series of analyses of HRV during different conditions: rest and sleep. RR interval data were extracted during each condition for the entire 3-day period. The output from the VitalPatch was downloaded as an Excel spreadsheet. The “Time” column was converted from UNIX epoch time to human-readable time. Next, the times on the logs for each condition (i.e., rest and sleep) were matched with the times on the RR interval output. The RR interval data for each condition was copied into a.txt file and was processed for HRV using Kubios HRV Premium software. Since the participant's physical activity performed during the tested days was not controlled, the HRV in this condition was not analyzed. However, considering the effects of physical activity on HRV (Felber Dietrich et al., 2008; Soares-Miranda et al., 2014), the total hand activity (*via* actigraphs) (Frey et al., 2022) and the total number of steps (Vista solution) were objectively quantified during the tested days.

**Table 1:** Time-domain results (3-day average: overall, sleep, rest) in ALS patients.

**Patient**	**Overall 3-day average**			**Sleep 3-day average**			**Rest 3-day average**		
	**pNN50**	**SDNN**	**RMSSD**	**pNN50**	**SDNN**	**RMSSD**	**pNN50**	**SDNN**	**RMSSD**
1	4.7	34.1	24.9	2.4	27.2	22.3	6.5	40.8	28.8
2	2.8	22.6	20.7	5.7	26.6	26.2	3.2	22.2	22.4
3	2.7	30.8	21.4	6.7	37.7	28.5	6.8	40.5	27.9
4	1.1	19.4	18.7	3.7	27.9	27.2	4.9	26.5	27.2
5	1.0	17.3	21.6	No data					
Mean ± SD	2.5 ± 1.5	24.5 ± 7.3	21.5 ± 2.3	4.6 ± 1.9	29.9 ± 5.3	26.0 ± 2.7	5.4 ± 1.7	32.5 ± 9.5	26.6 ± 2.9

### MRI Assessment of Insula

**Figure 3:**
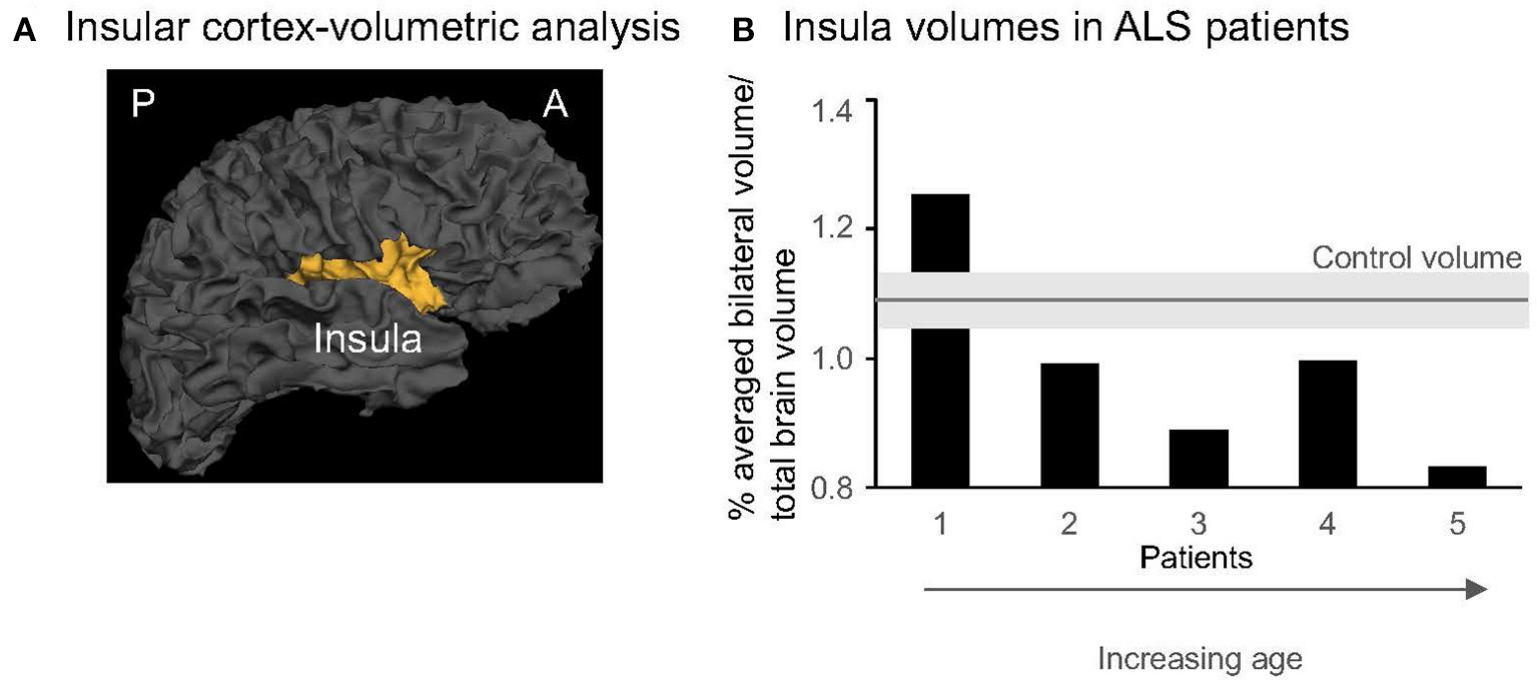
**(A)** 3-D gray matter insula in one control (Freesurfer, sagittal view). A, anterior; P, posterior. **(B)** Individual insular volumes were expressed as % of the total brain volume in patients (black columns) and controls (gray line indicates the mean, light gray rectangle indicates the SD for the group). The patients were presented from the youngest (1) to the oldest (5). Note that the last 3 patients (#3–5) are aged >50 years and represent the older subgroup.

MRI scans were performed on a Siemens 3T Prisma research-dedicated scanner with a 32-channel head coil. High-resolution (1 mm^3^) 3D structural T1-weighted MRI data was acquired and used to quantify the gray matter volume of the insula in each hemisphere (Figure 3A). Cortical reconstruction and volumetric segmentation were performed with the Freesurfer image analysis suite (http://surfer.nmr.mgh.harvard.edu/). The technical details are described in prior publications (Dale et al., 1999; Fischl et al., 2001, 2004; Desikan et al., 2006; Reuter et al., 2012). Briefly, this processing included motion correction and averaging of multiple volumetric T1 weighted images, removal of non-brain tissue using a hybrid watershed/surface deformation procedure, automated Talairach transformation, segmentation of the subcortical white matter and deep gray matter volumetric structures, intensity normalization, tessellation of the gray matter white matter boundary, automated topology correction, and surface deformation following intensity gradients to optimally place the gray/white and gray/cerebrospinal fluid borders at the location where the greatest shift in intensity defines the transition to the other tissue class. Once the cortical models were completed, a number of deformable procedures were performed, including surface inflation, registration to a spherical atlas which is based on individual cortical folding patterns to match cortical geometry across subjects, parcellation of the cerebral cortex into units with respect to gyral and sulcal structure, and creation of a variety of surface-based data including maps of curvature and sulcal depth. This method used both intensity and continuity information from the entire three-dimensional MR volume in segmentation and deformation procedures to produce representations of cortical thickness, calculated as the closest distance from the gray/white boundary to the gray/CSF boundary at each vertex on the tessellated surface. The maps were created using spatial intensity gradients across tissue classes and are therefore not simply reliant on absolute signal intensity. Additionally, the maps produced were not restricted to the voxel resolution of the original data thus are capable of detecting submillimeter differences between groups. Critically, we checked Freesurfer reconstructions and segmentations for quality control, and all segmentation edits were made *via* Freesurfer's Freeview GUI and then reprocessed *via* Freesurfer. For each participant, bilateral insular volumes were averaged and expressed as percent of the total intracranial volume.

### Statistical Analysis

Participant characteristics were summarized by mean (SD) for quantitative variables and by frequencies and proportions for qualitative variables. A *t*-test was used to assess between-group baseline differences in insular volume. Pearson rank-order correlation was used to determine the relationships between clinical, demographic, activity data, pNN50 values, and insular metrics. Significant level was considered at *p*-value < 0.05 (SPSS 23.0; SPSS Inc., Chicago, IL).

## Results

A high adherence rate for this type of continuous monitoring was found in all participants. Patients and relatives provided a series of positive feedback about their experience: enthusiastic use of such wearable devices, the feasibility of tablet return by mail, no feeling pressure from testing, and/or for transport for testing.

Our primary analyses investigating pNN50 for a total of 72h in real-world settings found an averaged pNN50 lower than 3% in three out of five patients (mean for the group, 2.49 ± 1.51%; Table 1). The subanalysis of sleep and rest conditions during the tested days demonstrated a lower pNN50 in one out of four patients for both conditions (4.61 ± 1.91% during sleep, 5.37 ± 1.65% during rest, Table 1). Similar to prior studies (Baltadzhieva et al., 2005; Pavlovic et al., 2010; Merico and Cavinato, 2011; Pinto et al., 2012; Pimentel et al., 2019), the pNN50 values in patients were not related to the clinical motor symptoms severity (as measured by ALSFRSr, *r* = 0.53, *p* = 0.4) or time after diagnosis (*r* = 0.33, *p* = 0.6). No correlations were found between pNN50 and anxiety level (*r* = 0.52, *p* = 0.4) or physical activity (steps, *p* = 0.3; right and left-hand activity, *p* = 0.9 and 0.8, respectively).

A lower 3-day averaged pNN50 was also found in three out of four controls (12.32 ± 21.14%). Although the controls were likely more active than patients during the tested days (right/left hand activity: 27.1 ± 5.7 h per 3 days/23.4 ± 6.9 h in controls, 13.2 ± 9.0 h/13.2 ± 8.8 h in patients, *p* = 0.12/0.25; # steps per 3 days: 26,413 ± 10,798 in controls, 9,015 ± 7,076 in ALS, *p* = 0.08), it is likely that the physical activity has no impact on HRV, at least, in this small sample.

Similar to the general population (Reardon and Malik, 1996; Umetani et al., 1998), negative correlations were found between pNN50 values and age in our patients (*r* = −0.95, *p* = 0.01) and controls (*r* = −0.91, *p* = 0.09). This might be reflective of reduced autonomic responsiveness to external environmental stimuli with age.

Our secondary analyses revealed no significant between-group differences in the insular gray matter volume across hemispheres (0.99 ± 0.16% in ALS vs. 1.09 ± 0.05% in controls, *p* = 0.26; Figure 3B). The insular volumes in patients were independent of clinical characteristics (disease duration, *r* = 0.18, *p* = 0.8; clinical severity, *r* = 0.48, *p* = 0.4, or anxiety, *r* = 0.86, *p* = 0.06) or 3-day pNN50 values (*r* = 0.74, *p* = 0.1). Yet, similar to pNN50, the insular volumes significantly decreased with aging (*r* = −0.91, *p* = 0.03). No such relationship was found in the control group (*r* = −0.86, *p* = 0.06), although the magnitude of the correlation coefficient indicates that a larger sample size would reach statistical significance. However, individual data showed that older patients (age > 50 years old, *n* = 3) have lower volumes than older matched controls (*n* = 3) (0.91 ± 0.09% in ALS vs. 1.11 ± 0.03%, *p* = 0.04, Figure 3B).

## Discussion

At present, no studies have investigated the feasibility of continuous monitoring of HRV in ALS patients. To our knowledge, this is the first pilot study examining a 3-day HRV profile in patients with ALS. This pilot study had several results: (i) no technical difficulties were reported; (ii) the wearable device was tolerated well by patients; (iii) testing duration was acceptable; and (iv) most of our patients displayed cardiovascular dysautonomia, as quantified by HRV, regardless of the disease's duration and severity. Secondarily, insula neurodegenerative processes in patients were similar to those in matched controls and not significantly related to HRV metrics in patients. These results are detailed below.

As previously reported (Baltadzhieva et al., 2005; Pavlovic et al., 2010; Merico and Cavinato, 2011; Pinto et al., 2012; Pimentel et al., 2019), our preliminary results demonstrated that three out of five patients exhibited sympathovagal imbalance. As mentioned above, HRV quantification was different between our study and the prior studies, a 3-day continuous quantification in real-world settings vs. standard autonomic tests consisted of a 1-min recording of heart rate during normal and deep breathing, Valsalva test, and heart rate response to active standing (one time in the lab). We did not find a correlation between the HRV indexes and the clinical features (measured *via* ALSFRS) or disease duration, indicating that the course of autonomic dysfunction does not move in parallel with motor neuron degeneration. Considering that similar findings were previously reported in these patients (Linden et al., 1998; Pavlovic et al., 2010; Merico and Cavinato, 2011), our preliminary data suggest that autonomic neuron degeneration may appear concomitant with the degeneration of motor neurons. However, due to the sample size, these results should be interpreted with caution. Nevertheless, our preliminary data illustrated the potential of this wearable device to provide continuous, unobstructed, and objective measures of HRV (and other parameters, such as step count, and respiratory rate) in real-world settings in ALS patients. The ability to assess such metrics of human behavior is a key prerequisite for precision medicine. If further work confirms these preliminary findings with larger sample size, such remote monitoring will have a major clinical impact by better fitting the physical abilities of these patients. Thus, patients will have the opportunity to participate in clinical trials from home, reducing the overall trial burden and the costs for long-term trials. Finally, the development of non-invasive, objective symptom monitoring approaches as an alternative to the survival-based endpoints currently used in clinical trials is also critically needed in this highly heterogeneous population where most therapies do not benefit from a one-size-fits-all approach.

Our secondary series of analyses revealed no significant differences in the insular volume, measured bilaterally, in patients vs. control participants. Yet, individual data showed a lower insular volume in older patients vs. older matched controls (Figure 3B). Are these data suggestive of accelerated neurodegenerative processes in this population? Due to the small sample size, we are very cautious in interpreting these data. Further work is warranted. Harnessing our imaging expertise (Cirstea et al., 2011, 2012, 2014, 2017, 2018), MR Spectroscopy will be used in future participants to examine insular microstructural changes. These changes could be detected in the early stages when structural MRI is unremarkable. For instance, decreased levels of an MR Spectroscopy-detected neuronal marker are considered an indicator of neuronal dysfunction before neuronal death occurs and a more sensitive indicator to neuronal death than gray matter structural metrics (Siegel et al., 2005) used in the current study. We did not find a significant correlation between the 3-day pNN50 values and insular volumes. However, the magnitude of the correlation coefficient indicates that a larger sample size would reach statistical significance. If our results will provide further support for such association, these findings will improve our understanding of the biological substrate of cardiovascular autonomic dysfunction in ALS.

### Study Limitations

The biggest limitation of this study is the small sample size (*n* = 5). However, the present study is pilot in nature and suggests the feasibility of real-world HRV measurement in patients with ALS. Further study with a large sample is required before any firm interpretation can be considered. We should also add that the patients capable of participating in research may be a distinct subtype as well, which could impact generalizability. This study is still ongoing and additional steps have been taken to increase patient recruitment. The procedures to mail the device/tablet/associated documentation have been already implemented in our lab. This followed by phone calls will allow us to extend the recruitment even outside of Missouri. Another limitation is the time after diagnosis; we now focus our efforts on recruiting only newly diagnosed patients. This is timely because there is an unprecedented need for such monitoring in ALS. It is possible that an increase in anxiety, which was not measured during the tested days, may partially explain our results. As such, the assessment of daily anxiety daily testing was added to the study protocol. For the second series of analyses, one limitation is the focus on only one brain area, the insula. We are aware of the difficulty to elucidate the precise central damage corresponding to the clinical autonomic manifestations and definitively future studies on other brain areas involved in cardiovascular autonomic control, e.g., the limbic system (Benarroch, 2012), are needed.

## Data Availability Statement

The original contributions presented in the study are included in the article/supplementary material; further inquiries can be directed to the corresponding author.

## Ethics Statement

The studies involving human participants were reviewed and approved by University of Missouri (MU) Human Subjects Review Board. The patients/participants provided their written informed consent to participate in this study.

## Author Contributions

AAB drafted the manuscript and performed MRI data acquisition and analysis. BJF performed HRV data processing and analysis and manuscript revision. VJ was responsible for patient recruitment. BEG, JDP, and IAA participated in patient recruitment, data acquisition, and analysis. DQB and RJB made critical revision of the manuscript for intellectual content. CMC conceived and designed the research, oversaw data acquisition and analysis, interpreted data, made critical revision of the manuscript for intellectual content, performed manuscript revision, handled funding, and supervision. All authors contributed to the article and approved the submitted version.

## Funding

This study was funded by Translational Research Impacting Useful and Meaningful Precision Health (TRIUMPH) Initiative Missouri-Columbia School of Medicine to CMC. BEG and JDP, both M1 medical students, were supported by the Summer Research Fellowship Program at the University of Missouri-Columbia School of Medicine.

## Conflict of Interest

The authors declare that the research was conducted in the absence of any commercial or financial relationships that could be construed as a potential conflict of interest.

## Publisher's Note

All claims expressed in this article are solely those of the authors and do not necessarily represent those of their affiliated organizations, or those of the publisher, the editors and the reviewers. Any product that may be evaluated in this article, or claim that may be made by its manufacturer, is not guaranteed or endorsed by the publisher.

## Abbreviations

ALS: Amyotrophic lateral sclerosis
ALSFRS-R: Revised ALS Functional Rating Scale
ANS: Autonomic nervous system
CSF: Cerebrospinal fluid
HRV: Heart rate variability
min: minute
ms: milliseconds
pNN50: Percentage of successive RR intervals that differ by more than 50 ms
SD: standard deviation.
